# Sequential scans of ^18^F-flutemetamol PET using PET/CT and PET/MRI: influence of amyloid burden, scan interval, and age

**DOI:** 10.1007/s11604-025-01915-1

**Published:** 2025-12-10

**Authors:** Shin Morooka, Yasutaka Fushimi, Sachi Okuchi, Akihiko Sakata, Takayuki Yamamoto, Satoshi Nakajima, Katsuhiko Mitsumoto, Koji Itagaki, Manabu Kubota, Atsushi Shima, Sakiho Ueda, Kazuya Goto, Akira Kuzuya, Takashi Hanakawa, Nobukatsu Sawamoto, Yuji Nakamoto

**Affiliations:** 1 Department of Diagnostic Imaging and Nuclear Medicine, Graduate School of Medicine, Kyoto University, Kyoto, Japan; 2 Department of Clinical Radiology Service, Kyoto University Hospital, Kyoto, Japan; 3 Department of Psychiatry, Graduate School of Medicine, Kyoto University, Kyoto, Japan; 4 Department of Human Health Sciences, Graduate School of Medicine, Kyoto University, Kyoto, Japan; 5 Department of Neurology, Graduate School of Medicine, Kyoto University, Kyoto, Japan; 6 Department of Regulation of Neurocognitive Disorders, Graduate School of Medicine, Kyoto University, Kyoto, Japan; 7 Department of Integrated Neuroanatomy and Neuroimaging, Graduate School of Medicine, Kyoto University, Kyoto, Japan; 8 Human Brain Research Center, Graduate School of Medicine, Kyoto University, Kyoto, Japan; 9 Biomedical Imaging Research Center, University of Fukui, Eihieijicho, Japan

**Keywords:** Amyloid, ^18^F-flutemetamol, Centiloid scale, Standardized uptake value ratio

## Abstract

**Purpose:**

We aimed to compare visual and quantitative assessments between PET/CT at 90 min and sequential PET/MRI at approximately 120 min after injection of ^18^F-flutemetamol, and to investigate factors affecting differences in standardized uptake value ratio (SUVr) and Centiloid scale.

**Materials and methods:**

Eighty-three participants underwent both PET/CT and sequential PET/MRI. Two nuclear medicine physicians performed visual interpretations. SUVr was calculated using four reference regions—pons, whole cerebellum, cerebellar gray matter (CGM), and whole cerebellum and brainstem—and compared between PET/CT and PET/MRI. Centiloid Scale was also compared between PET/CT and PET/MRI. Subgroup analyses were conducted based on Centiloid scale. Associations between scan interval, age, and amyloid burden were evaluated using stepwise regression. Cerebrospinal fluid (CSF) biomarkers were compared with imaging findings in 52 participants. Additionally, six participants underwent dynamic PET/MRI at 0, 60, 90, and 120 min post-injection.

**Results:**

Visual interpretation showed high agreement between PET/CT and PET/MRI (κ = 0.97), and 98% concordance with CSF findings. SUVr and Centiloid scale demonstrated a strong intraclass correlation coefficient of 0.96–0.98. However, SUVr and Centiloid scale were significantly higher from PET/CT than from PET/MRI, except for SUVr using CGM. Age and Centiloid scale were significant predictors of modality differences. Scan interval was also significant when CGM was used as the reference region. Dynamic PET/MRI revealed time-dependent increases in Centiloid scale among amyloid-positive participants.

High concordance in visual and quantitative assessments was seen for ^18^F-flutemetamol PET/CT (90 min) and subsequent PET/MRI (120 min). While diagnostic agreement was preserved, quantitative values were influenced by amyloid burden, age, and scan timing. These findings suggest a need for careful consideration when interpreting quantitative metrics across different imaging modalities and time points.

**Supplementary Information:**

The online version contains supplementary material available at 10.1007/s11604-025-01915-1.

## Background

Amyloid-beta (Aβ) accumulation and tau pathologies in the brain are characteristic of Alzheimer’s disease (AD) [1, 2], and demonstration of amyloid burden on either amyloid PET or cerebrospinal fluid (CSF) biomarkers is necessary for anti-Aβ treatments [3, 4]. Visual interpretation of whether the result for amyloid is positive or negative is performed in clinical settings, since visual interpretation of amyloid PET allows in vivo detection of Aβ in the brain with high sensitivity and specificity [5–8], and a negative result from an amyloid PET scan reflects a reduced likelihood of AD [2, 9]. Quantitative values such as Centiloid scale and standardized uptake value ratio (SUVr) are often used, but should be interpreted with caution, as factors such as scanner type, imaging protocol, image reconstruction, software, and post-processing procedures can all influence the results [10, 11].

Specific criteria for amyloid PET image visual interpretation differ among PET tracers, and visual interpretation should be performed using the instructions provided by the manufacturers with the following amyloid PET imaging guidelines [12, 13]. According to the standard protocol for ^18^F-flutemetamol PET, the start time of the PET scan is 60–120 min after ^18^F-flutemetamol injection, and an acquisition time of 20–30 min is recommended. Most amyloid PET research adheres to this imaging protocol with PET/CT; however, there are disagreements rates around 10% between visual ratings and global SUVr [14], between raters [15], visually assessed as equivocal around 23% [16], and between visual interpretation and quantitative assessment around 5.4% [17]. As indicated in a phase I study of ^18^F-flutemetamol PET, delayed-phase imaging may indicate a greater cumulative increase in SUVr in amyloid-positive groups than in amyloid-negative groups [18]. To the best of our knowledge, no comparative study of ^18^F-flutemetamol PET between recommended and delayed phases has been published. In addition, visual interpretation and the Centiloid scale of sequential PET scans have not been investigated thoroughly.

Recently, PET/MRI has also been used for amyloid PET with ^18^F-flutemetamol [19–24]. While PET/CT is commonly used for amyloid imaging, PET/MRI has been introduced into clinical practice due to the advantages such as superior soft tissue contrast, reduced radiation exposure, and simultaneous acquisition of PET and MRI data. Compared with PET/CT, bone tissue cannot easily be imaged and may be misclassified on PET/MRI. However, this limitation has been overcome through MR-based attenuation correction using a zero echo time (ZTE) sequence [25]. Although the inclusion of amyloid PET/MRI in multicenter studies remains uncommon due to its limited availability and higher operational costs compared to PET/CT [17], its implementation can offer substantial benefits in certain research contexts. Further, one recent paper using ^18^F-florbetaben compared PET/CT at 70 min post-injection and sequentially performed PET/MRI at 90 min post-injection, revealing that SUVr was significantly higher from PET/MRI than from PET/CT [26]. Although there are differences of imaging modalities between PET/CT and PET/MRI, including the detectors (photomultiplier tube for PET/CT; semiconductor detector for PET/MRI), the effect of post-injection time should be clarified.

We hypothesized that the delayed phase of ^18^F-flutemetamol PET would have minimal influence on visual interpretation for diagnosis, but might show differences in quantitative values between amyloid-positive and -negative cases, possibly due to differences in the clearance of the PET tracer from the cerebral cortices. Although PET/CT was selected based on the pre-established clinical research protocol, PET/MRI could be subsequently incorporated as an additional imaging modality [26]. This study aimed to qualitatively and quantitatively evaluate sequential ^18^F-flutemetamol PET/CT and PET/MRI scans among participants enrolled under the initial PET/CT clinical protocol.

## Methods

### Study participants

Participants scheduled to undergo amyloid PET/CT as part of ongoing clinical research were recruited prospectively between June 2020 and September 2023. The studies included in the ongoing research protocol were the Parkinson’s and Alzheimer’s disease Dimensional Neuroimaging Initiative (PADNI) study (UMIN000036297) and clinical research protocol C1546, which also targeted amyloid PET for mild cognitive impairment (MCI), Parkinson’s disease (PD), depression, and healthy controls. The local institutional review board approved this study, and all participants provided written informed consent. The study cohort comprised 84 individuals (76 from PADNI, and 8 from C1546): 22 with clinical MCI, 37 with PD, 3 with PD with dementia, 7 with depression, and 15 healthy controls. Following diagnostic criteria was used for the participants: MCI due to AD, the clinical diagnostic criteria of probable AD dementia and MCI due to AD based on the 2011 guidelines in the National Institute on Aging-Alzheimer’s Association [27]; PD, MDS clinical diagnostic criteria for PD [28], depression, the Diagnostic and Statistical Manual of Mental Disorders Fifth Edition (DSM-5) [29]. However, one participant (with PD) was excluded from the study because the tracer dose differed from the standard dose, resulting in results from a total of 83 participants being analyzed in this study (Supplementary Fig. 1).

### Amyloid PET

All participants received an intravenous injection of ^18^F-flutemetamol (185 MBq; Vizamil, Nihon Medi-Physics Co., Tokyo, Japan). PET/CT was performed 90 min post-injection with an acquisition duration of 20 min, followed by sequential PET/MRI.

(i) The PET/CT protocol was as follows. A PET/CT scanner equipped with photomultiplier tube (PMT)-based detectors (Discovery IQ; GE Healthcare, Milwaukee, WI, USA) reconstructed with VUE Point HD (3D ordered subset expectation maximization method, OSEM), subset of 12, iteration times of 5, and filter cutoff of 4.0 mm was applied. Field of view (FOV) was 25 cm, matrix size was 128 × 128, and voxel size was 1.95 × 1.95 × 3.26 mm^3^.
(ii) The PET/MRI protocol was as follows. A 3-T PET/MRI scanner equipped with silicon photomultiplier (SiPM)–based time-of-flight (TOF) detectors (Signa PET/MR; GE Healthcare, Waukesha, WI, USA) with a 19-channel HNU coil was used. PET data acquisition employed the 3D and list modes for 20 min per bed position, covering 89 slices. Image reconstruction incorporated time-of-flight and 3D OSEM with 16 subsets and four iterations, followed by a 4-mm Gaussian filter post-smoothing, resulting in a 128 × 128 matrix size. MR attenuation correction was conducted using two methods: a 2-point Dixon 3D T1-weighted fast SPGR sequence (repetition time/echo time (TE) 1/TE2, 4.0/1.1/2.2 ms; FOV, 50 × 37.5 cm; matrix, 256 × 128; slice thickness/spacing, 5.2/2.6 mm; 120 images/slab; acquisition time, 18 s) and a ZTE sequence for bone identification.

### Visual interpretation

Two board-certified nuclear medicine physicians (S.M., Y.F.), both specialized in neuroradiology and trained in mandatory amyloid PET interpretation, independently reviewed the amyloid PET images. Each rater evaluated the amyloid PET/MRI images first, and after a 3-week interval, assessed the amyloid PET/CT images, categorizing them as either positive or negative for amyloid accumulation following established guidelines.

In cases where the interpretations of raters differed, the raters conducted a collaborative review of the findings to reach a consensus on the final classification. This approach was employed to ensure consistency in the visual interpretation of amyloid PET imaging.

### Quantitative analysis

Measurement of pixel values and calculation of SUVr and Centiloid scale were performed using VIZCalc, which is included in medi + FALCON version 1.3 (Nihon Medi-Physics Co., Tokyo, Japan). VIZCalc is equipped with an anatomical standardization function and volume of interests (VOIs) for Centiloid scale calculation: the cerebral cortex (Cortex), pons, whole cerebellum (WC), cerebellar gray matter (CGM), and whole cerebellum and brainstem (WCB). One participant was excluded from the quantitative analysis due to an artifact caused by a metal strip in the face mask, which led to inaccurate attenuation correction in PET/MRI. As a result, data from 82 participants were included in the final quantitative analysis. Importantly, this metallic artifact did not affect PET images for visual interpretation.

Quantitative values, SUVr derived from each reference region, SUVr (pons), SUVr (WC), SUVr (CGM), SUVr (WCB), and the Centiloid scale (calculated from the WC) were compared between PET/CT and PET/MRI in all participants, as well as between those with low Centiloid scale values and those with high Centiloid scale values. We adopted the cutoff value of Centiloid scale at 20, since previous studies have used thresholds ranging from 12 to 30 [10, 17]) and one large trial defined intermediate amyloid burden as 20 to 40 Centiloid scale and elevated amyloid burden as > 40 Centiloid scale [30].

To evaluate the trend between PET/CT and sequential PET/MRI, ratios of SUVr values obtained from PET/CT to PET/MRI ((SUVr_PET/CT)/(SUVr_PET/MRI) ratio) were compared in all participants, as well as between those with low Centiloid scale values (< 20) and those with high Centiloid scale values (≥ 20). Centiloid scale obtained from PET/CT to PET/MRI ((Centiloid_PET/CT)—(Centiloid_PET/MRI)) was compared in the same manner. Centiloid differences ((Centiloid_PET/CT)—(Centiloid_PET/MRI)) were expressed as absolute values, because the Centiloid scale may take negative numbers.

### CSF analysis

CSF analysis was performed in 53 of the 83 participants. One case was excluded because of specimen-related issues that made the evaluation difficult. The clinical diagnosis in cases where CSF analysis was performed was 18 with MCI, 21 with PD, 2 with PD with dementia, and 12 with healthy control. The CSF analysis was performed at a median of − 14 days [− 1152, 791] compared to the PET examination date. CSF samples were collected in sterile polypropylene tubes by lumbar puncture, then aliquoted in polypropylene vials at – 80 °C until analysis. Aβ40 and Aβ42 were measured using a Human β Amyloid (1–40) ELISA Kit Wako II (FUJIFILM Wako Pure Chemical Corporation, Osaka, Japan) and Human β Amyloid (1–42) ELISA Kit Wako, High Sensitive (FUJIFILM Wako Pure Chemical Corporation). All analyses were performed in accordance with the instructions from the manufacturer. Aβ40/Aβ42 ratio was classified as positive or negative using a cutoff value of 12 [31–33].

### Dynamic amyloid PET/MRI

Since no participants underwent PET/MRI at 90 min post-injection or PET/CT at approximately 120 min post-injection, differences in PET scanners between PET/CT and PET/MRI may have affected the results. To assess the impact of this confounding factor, we recruited six participants who had previously undergone amyloid assessment (three amyloid-positive and three amyloid-negative cases) for dynamic amyloid PET/MRI. All participants underwent four consecutive amyloid PET/MRI immediately after injection of ^18^F-flutemetamol (approximately 185 MBq) at time points of 0, 60, 90, and 120 min post-injection. Each PET acquisition lasted 15–20 min. After the first scan of 0–15 (or 20) min, participants rested. MR-based attenuation correction was performed for each scan.

SUVr with four reference regions (pons, WC, CGM, and WCB) and the Centiloid scale were calculated for PET/MRI data at 0, 60, 90, and 120 min post-injection.

### Statistical analyses

The inter-rater agreement of visual interpretations of amyloid PET imaging was assessed using Cohen’s κ coefficient. The consistency of the SUVr and Centiloid scale between PET/CT and PET/MRI was evaluated using intraclass correlation coefficient (ICC). Paired t-tests were used for quantitative values comparisons between PET/CT and PET/MRI in all participants. Unpaired t-tests were used for comparison between low and high Centiloid scale groups because the differences were normally distributed according to Shapiro–Wilk on the difference scores.

Spearman’s rho analysis was used for univariate analyses of (SUVr_PET/CT)/(SUVr_PET/MRI) ratio, since normal distributions were not observed for variables. We performed a stepwise multiple linear regression analysis to identify factors associated with (SUVr_PET/CT)/(SUVr_PET/MRI) ratio. The selection criterion was based on p-values, with a threshold of 0.25 for both entry and removal. A bidirectional stepwise approach was used, allowing variables to be added or removed iteratively based on the statistical contribution to the model.

All statistical analyses were performed using JMP Pro version 18.0 software (SAS Institute Inc., Cary, NC, USA).

Correlations of (SUVr_PET/CT)/(SUVr_PET/MRI) ratio with Centiloid scale, age, sex, and the interval between PET/CT and PET/MRI (scan interval) were evaluated using univariate analyses. Subsequently, multiple regression analysis was performed using the (SUVr_PET/CT)/(SUVr_PET/MRI) ratio as the dependent variable, with independent variables selected from Centiloid scale, age, sex, and scan interval, if the p-value was less than 0.20. In addition, another multiple regression analysis was also performed using (Centiloid_PET/CT)—(Centiloid_PET/MRI) as the dependent variable and the same set of independent variables.

## Results

In total, 83 participants were enrolled for visual interpretation (median age: 71.0 years; interquartile range, 65.0–75.0 years; 42 females, 41 males). The median injected dose of ^18^F-flutemetamol for all participants (n = 83) was 189.0 MBq (interquartile range, 186.0–191.8 MBq). One participant (a 76-year-old male) was excluded due to the metallic artifact associated with the face mask, resulting in 82 participants (median age, 71.0 years; interquartile range, 65.0–75.0 years; 42 females, 40 males) being enrolled for quantitative analysis. The median scan interval between PET/CT and PET/MRI scans was 32.8 min (interquartile range, 30.7–36.8 min).

### Visual interpretation

Table 1 presents the inter-rater agreement of visual interpretation of amyloid PET/CT and PET/MRI. Inter-rater disagreements were observed in 4 cases of PET/CT and 3 cases of PET/MRI. The calculated κ coefficients were 0.88 for PET/CT and 0.91 for PET/MRI. In most cases, evaluations of PET/CT and PET/MRI demonstrated concordance, except for 4 cases for Rater A and 3 cases for Rater B. The calculated κ coefficients were 0.85 for Rater A and 0.88 for Rater B.

**Table 1:** Inter-rater agreement between Rater A and Rater B for PET/CT and PET/MRI

		Rater A	
		Negative	Positive
*PET/CT*			
Rater B	Negative	57	2
	Positive	2	22
*PET/MRI*			
Rater B	Negative	59	3
	Positive	0	21

Upon reaching consensus for raters, a case of discrepancy was identified between PET/CT and PET/MRI, resulting in a κ coefficient of 0.97 (Table 2). Despite the scan interval between tracer administration and imaging, PET/MRI exhibited high concordance with PET/CT in terms of visual interpretation.

**Table 2:** Agreement in visual interpretation between PET/CT and PET/MRI

		PET/CT	
		Negative	Positive
PET/MRI	Negative	61	1
	Positive	0	21

For cases visually classified as negative, Centiloid values ranged from − 18 to 26.9 for PET/CT and − 25.7 to 25.1 for PET/MRI. In contrast, visually positive cases exhibited a generally higher Centiloid scale, with PET/CT values ranging from 30.8 to 119.2 and PET/MRI values from 49.4 to 122.4. Among the cases with visually interpreted as positive on PET/CT, the one with the lowest Centiloid scale who was visually assessed as negative on PET/MRI was a Centiloid scale of 22.1 on PET/CT. Conversely, the case with visually interpreted as positive case on PET/MRI was also interpreted as positive on PET/CT, having a Centiloid scale of 44.9 on PET/MRI.

### SUVr and Centiloid scale consistency and ICC

**Fig. 1:**
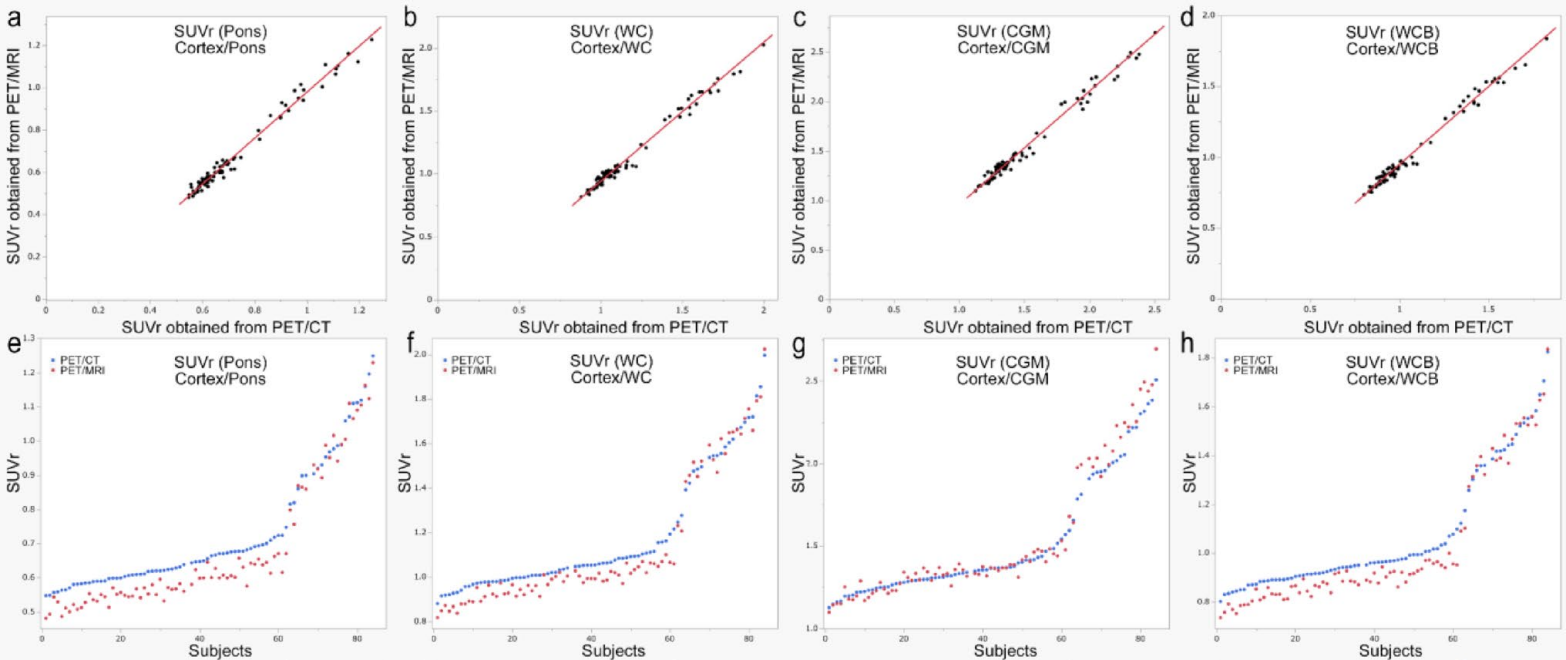
SUVr values obtained from both PET/CT and PET/MRI. **a** SUVr (Pons), i.e., cortex/pons; **b** SUVr (Cortex/WC), i.e., Cortex/WC; **c** SUVr (Cortex/CGM), i.e., Cortex/CGM; and **d** SUVr (Cortex/WCB), i.e., Cortex/WCB. Subjects are plotted in the order of increasing SUVr values obtained from PET/CT as follows: **e** SUVr (Pons), i.e., cortex/pons; **f** SUVr (Cortex/WC), i.e., Cortex/WC; **g** SUVr (Cortex/CGM), i.e., Cortex/CGM; and **h** SUVr (Cortex/WCB), i.e., Cortex/WCB. Blue circles represent PET/CT, and red circles represent PET/MRI, respectively. WC, whole cerebellum; CGM, cerebellar gray matter; WCB, whole cerebellum and brainstem

The median and range of SUV in each region are shown in Supplementary Table 1. SUVr values obtained from both PET/CT and PET/MRI are shown by reference region in Fig. 1. Participants are arranged in order of increasing SUVr values from PET/CT for each reference region in Fig. 1.

**Fig. 2:**
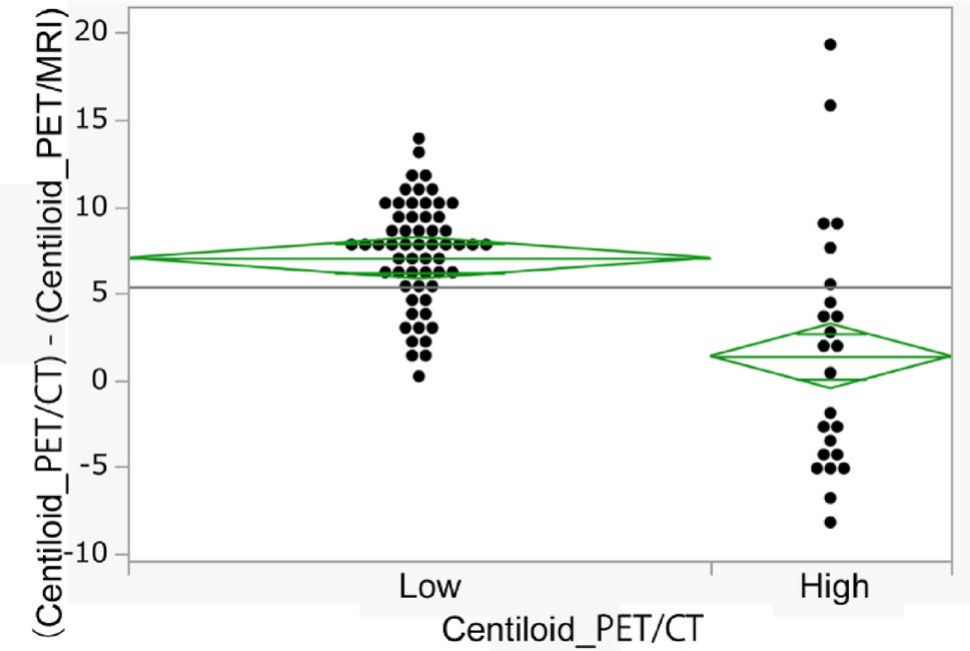
Centiloid scale of both PET/CT and PET/MRI, differences between Centiloid scale from PET/CT and PET/MRI. **a** Centiloid scale values obtained from both PET/CT (Centiloid_PET/CT) and PET/MRI (Centiloid_PET/MRI) are plotted, showing a high correlation (R^2^ = 0.99, *p* < 0.001). b Differences between Centiloid scale values from PET/CT and PET/MRI ((Centiloid_PET/CT)—(Centiloid_PET/MRI)) are compared against Centiloid_PET/CT. Notably, the trend of these differences varies depending on Centiloid scale values, with a moderate correlation (R^2^ = 0.30, *p* < 0.001)

Table 3 shows the results of SUVr values and the Centiloid scale obtained from both PET/CT and PET/MRI. Figure 2 shows plots of the Centiloid scale at both scans. SUVr and the Centiloid scale obtained from PET/CT were significantly higher than those obtained from PET/MRI, except SUVr (CGM), which was significantly lower on PET/CT (Table 3).

**Table 3:** SUVr values by reference regions and Centiloid scale

	All participants (n = 82)			Low Centiloid scale (< 20) (n = 58)			High Centiloid scale (≥ 20) (n = 24)		
	PET/CT	PET/MRI	*p*-value	PET/CT	PET/MRI	*p*-value	PET/CT	PET/MRI	*p*-value
SUVr (Pons)	0.66 [0.55, 1.25]	0.60 [0.48, 1.23]	< 0.001*	0.62 [0.55, 0.75]	0.57 [0.48, 0.67]	< 0.001*	0.96 [0.71, 1.25]	0.95 [0.61, 1.23]	0.006*
SUVr (WC)	1.06 [0.88, 2.00]	1.01 [0.82, 2.02]	< 0.001*	1.02 [0.88, 1.17]	0.97 [0.82, 1.10]	< 0.001*	1.55 [1.19, 2.00]	1.57 [1.06, 2.02]	0.35
SUVr (CGM)	1.37 [1.13, 2.51]	1.37 [1.10, 2.69]	< 0.001*	1.31 [1.13, 1.52]	1.33 [1.10, 1.48]	0.90	2.00 [1.54, 2.51]	2.09 [1.48, 2.69]	< 0.001*
SUVr (WCB)	0.96 [0.80, 1.82]	0.92 [0.74, 1.84]	< 0.001*	0.93 [0.80, 1.07]	0.88 [0.74, 1.00]	< 0.001*	1.42 [1.08, 1/82]	1.41 [0.95, 1.83]	0.11
Centiloid scale	4.2 [− 18, 119.2]	− 2.1 [− 25.7, 122.4]	< 0.001*	− 1.15 [− 18, 16.6]	− 6.5 [− 25.7, 8.9]	< 0.001*	64.3 [20.4, 119.2]	67.1 [3.9, 122.4]	0.35

With SUVr, regions in parentheses represent the corresponding reference region. Centiloid scale was calculated from SUVr (whole cerebellum). Low Centiloid scale (< 20) and high Centiloid scale (≥ 20) groups are divided according to the Centiloid scale obtained from PET/CT. The median and range of each SUVr value or Centiloid scale are presented. Mean value of SUVr (CGM) for all participants was 1.52 for PET/CT and 1.53 for PET/MRI

WC, whole cerebellum; CGM, cerebellar gray matter; WCB, whole cerebellum and brainstem

^*^Statistical significance

We also compared the low Centiloid scale group (71 years [63, 74], 29 males, 29 females) and high Centiloid scale group (75 years [67, 77], 13 females, 11 males). No significant differences in age or sex were observed between groups. In the low Centiloid scale group, SUVr and Centiloid scale obtained from PET/CT were significantly higher than those obtained from PET/MRI, again except for SUVr (CGM), which was lower. In contrast, the high Centiloid scale group showed different trends: SUVr (Pons) was significantly higher on PET/CT, while SUVr (CGM) remained significantly lower on PET/CT. No other metrics showed significant differences.

A high ICC of 0.96–0.97 was observed for SUVr between PET/CT and PET/MRI (Table 4). Regarding the Centiloid scale, PET/CT yielded a median of 4.2 [− 18.0, 119.2], while PET/MRI demonstrated a median of − 2.1 [− 25.7, 122.4], with an ICC of 0.98 [0.82, 0.99] (Table 4). The high ICC values observed for both SUVr and Centiloid scale indicate that PET/CT and PET/MRI provide consistent and reliable measures, notwithstanding variations in imaging times between the two modalities.

**Table 4:** ICC of SUVr between PET/CT and PET/MRI

	ICC
SUVr (Pons)	0.96 [0.32, 0.99]
SUVr (WC)	0.98 [0.82, 0.99]
SUVr (CGM)	0.98 [0.97, 0.99]
SUVr (WCB)	0.97 [0.67, 0.99]
Centiloid scale	0.98 [0.82, 0.99]

With SUVr, regions in parentheses represent the corresponding reference region. Centiloid scale was calculated from SUVr (whole cerebellum). Values in square brackets represent 95% confidence intervals (CIs)

WC, whole cerebellum; CGM, cerebellar gray matter; WCB, whole cerebellum and brainstem

Bland–Altman plots were presented in Supplemental Figs. 4 and 5. The Bland–Altman plot demonstrated that Centiloid Scale and SUVr values between PET/CT and PET/MRI were mostly within ± 1.96 standard deviations.

### Comparison of visual interpretation, CSF analysis, and Centiloid scale

CSF analysis was performed for total 52 participants. Visual interpretation of amyloid PET imaging, CSF analysis of amyloid, and Centiloid scale obtained from PET/CT and PET/MRI were compared, and CSF analysis revealed 17 amyloid-positive and 35 amyloid-negative cases. Visual interpretation of PET/CT or PET/MRI was concordant with CSF analysis in all but one case. In that single discordant case, a CSF amyloid-positive case was visually interpreted as amyloid-negative from both PET/CT and PET/MRI. In that case, Centiloid scale values were 26.9 on PET/CT and 25.1 on PET/MRI. In the CSF amyloid-positive group (n = 16, one case was excluded from analysis because of inaccurate SUV measurements), mean Centiloid scale was 71.8 ± 20.8 for PET/CT and 71.5 ± 21.4 for PET/MRI. Conversely, the CSF amyloid-negative group (n = 35) exhibited a mean Centiloid scale of 0.86 ± 2.26 on PET/CT and − 6.45 ± 7.47 on PET/MRI.

### (SUVr_PET/CT)/(SUVr_PET/MRI) ratio and Centiloid scale groups

The (SUVr_PET/CT)/(SUVr_PET/MRI) ratios of four reference regions are presented for all participants, and for low Centiloid scale (< 20) and high Centiloid scale (≥ 20) groups (Table 5). A ratio of (SUVr_PET/CT)/(SUVr_PET/MRI) greater than 1 indicates that the SUVr obtained from PET/CT was higher than the corresponding SUVr from PET/MRI, whereas a ratio less than 1 indicates the opposite. The (SUVr_PET/CT)/(SUVr_PET/MRI) ratios in the low Centiloid scale (< 20) group were significantly higher than those in the high Centiloid scale (≥ 20) groups when using four reference regions (pons, WC, CGM, and WCB) (*p* = 0.01, 0.001, 0.03, and 0.001, respectively).

**Table 5:** Ratios of SUVr values obtained from PET/CT to PET/MRI ((SUVr_PET/CT)/(SUVr_PET/MRI)) ratio

(SUVr_PET/CT)/(SUVr_PET/MRI) ratio	All participants (n = 82)	Low Centiloid scale (< 20) (n = 58)	High Centiloid scale (≥ 20) (n = 24)
Ratios of SUVr (Pons)	1.08 [0.96, 1.18]	1.10 [1.02, 1.18]	1.02 [0.96, 1.18]
Ratios of SUVr (WC)	1.05 [0.96, 1.15]	1.06 [1.00, 1.12]	1.01 [0.96, 1.15]
Ratios of SUVr (CGM)	0.99 [0.90, 1.07]	1.00 [0.95, 1.07]	0.95 [0.90, 1.06]
Ratios of SUVr (WCB)	1.06 [0.96, 1.15]	1.07 [1.02, 1.13]	1.01 [0.96, 1.15]

**Fig. 3:**
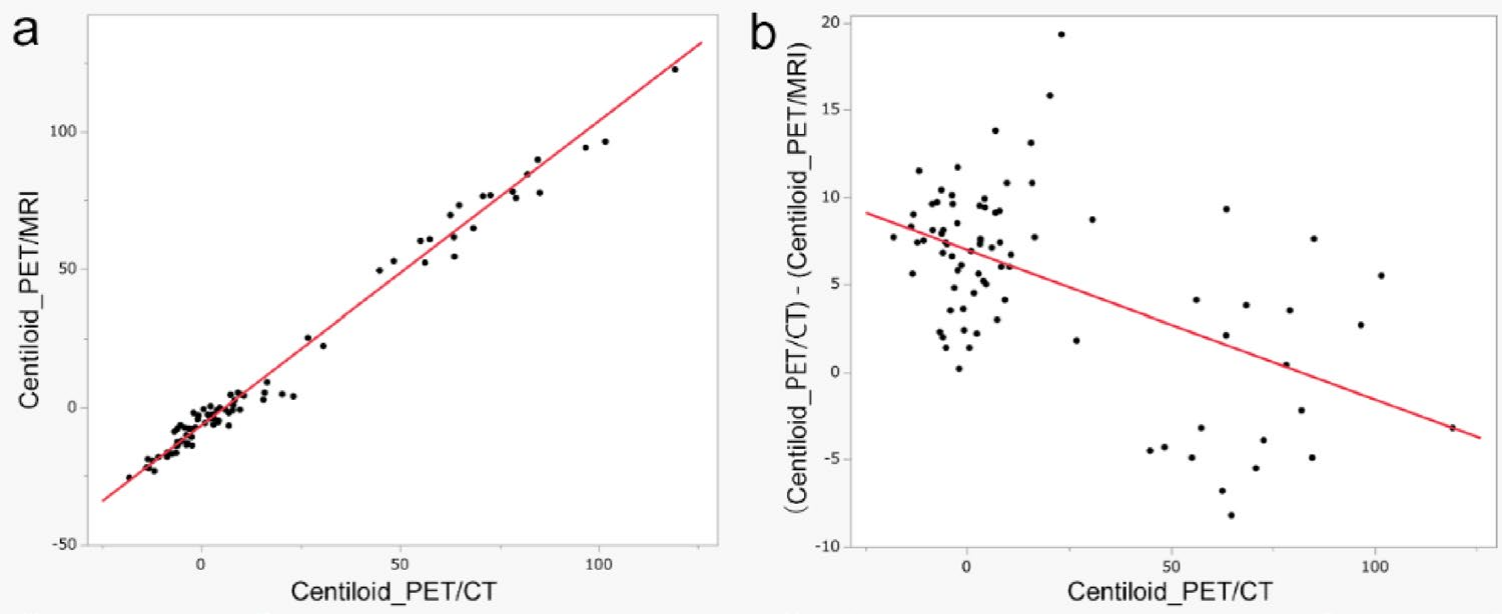
Differences between Centiloid scale values across low and high Centiloid scale groups. Differences between Centiloid scale values from PET/CT and PET/MRI ((Centiloid_PET/CT)—(Centiloid_PET/MRI)) are compared between low Centiloid scale (< 20) and high Centiloid scale (≥ 20). (Centiloid_PET/CT)—(Centiloid_PET/MRI) is significantly higher for low Centiloid scale (7.03 ± 3.00) than for high Centiloid scale (1.38 ± 7.09) (*p* < 0.001)

This table summarizes plotted data in Fig. 3. With SUVr, regions in parentheses represent the corresponding reference region. Centiloid scale was calculated from SUVr (WC). Low Centiloid scale (< 20) and high Centiloid scale (≥ 20) groups are divided according to Centiloid scale obtained from PET/CT. The median and range of each SUVr value or Centiloid scale are presented

(SUVr_PET/CT)/(SUVr_PET/MRI) ratio greater than 1 indicates that the SUVr obtained from PET/CT is higher than the corresponding SUVr from PET/MRI, whereas a ratio less than 1 indicates the opposite

WC, whole cerebellum; CGM, cerebellar gray matter; WCB, whole cerebellum and brainstem

(Centiloid_PET/CT)—(Centiloid_PET/MRI) were compared between the low and high Centiloid scale values group using the cutoff value 20 (Fig. 3). The difference in Centiloid scale between PET/CT and PET/MRI was 7.4 [0.2, 13.8] for the low Centiloid group and 1.1 [− 8.2, 19.3] for the high Centiloid group (*p* < 0.001).

### Univariate analysis of (SUVr_PET/CT)/(SUVr_PET/MRI) ratio and (Centiloid_PET/CT)—(Centiloid_PET/MRI)

**Fig. 4:**
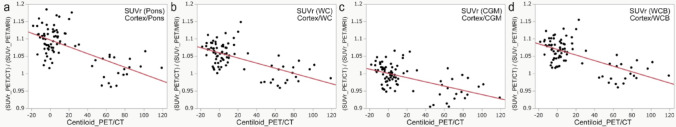
Relationship between ((SUVr_PET/CT)/(SUVr_PET/MRI)) ratio and Centiloid_PET/CT scale obtained from PET/CT (Centiloid_PET/CT). **a** SUVr using the pons as the reference region (Cortex/Pons); **b** SUVr using WC as the reference region (Cortex/WC); **c** SUVr using the CGM as the reference region (Cortex/CGM); and **d** SUVr using WCB as the reference region (Cortex/WCB). All correlations are statistically significant (*p* < 0.001), with correlation coefficients (ρ) of − 0.49, − 0.52, − 0.51, and − 0.52, respectively (Table 6). WC, whole cerebellum; CGM, cerebellar gray matter; WCB, whole cerebellum and brainstem

Spearman’s rho analysis was used for univariate analyses of (SUVr_PET/CT)/(SUVr_PET/MRI) ratio, since a normal distribution was not observed for variables. A significant negative correlation was demonstrated between Centiloid scale and (SUVr_PET/CT)/(SUVr_PET/MRI) ratios of all reference regions (pons, WC, CGM, and WCB; − 0.49, − 0.52, − 0.51, and − 0.52, respectively) (Fig. 4, Table 6). A significant correlation was demonstrated between scan interval and (SUVr_PET/CT)/(SUVr_PET/MRI) ratio (CGM) only (ρ = − 0.29, *p* = 0.01) (Supplementary Fig. 2, Table 6). A correlation was suggested between age and (SUVr_PET/CT)/(SUVr_PET/MRI) ratio (CGM) (ρ = 0.19, *p* = 0.09) (Supplementary Fig. 3, Table 6). No difference was seen between sex and (SUVr_PET/CT)/(SUVr_PET/MRI) ratios.

**Table 6:** Univariate analyses of ((SUVr_PET/CT)/(SUVr_PET/MRI)) ratio and (Centiloid_PET/CT)—(Centiloid_PET/MRI)

	Centiloid scale	Scan interval	Age	Sex
(SUVr_PET/CT)/(SUVr_PET/MRI) (Pons)	ρ = − 0.49 (*p* < 0.001)*	ρ = − 0.16 (*p* = 0.16)	ρ = 0.05 (*p* = 0.68)	*p* = 0.85
(SUVr_PET/CT)/(SUVr_PET/MRI) (WC)	ρ = − 0.52 (*p* < 0.001)*	ρ = − 0.17 (*p* = 0.13)	ρ = 0.10 (*p* = 0.36)	*p* = 0.91
(SUVr_PET/CT)/(SUVr_PET/MRI) (CGM)	ρ = − 0.51 (*p* < 0.001)*	ρ = − 0.29 (*p* = 0.01)*	ρ = 0.19 (*p* = 0.09)	*p* = 1.00
(SUVr_PET/CT)/(SUVr_PET/MRI) (WCB)	ρ = − 0.52 (*p* < 0.001)*	ρ = − 0.17 (*p* = 0.13)	ρ = 0.09 (*p* = 0.44)	*p* = 0.95
(Centiloid_PET/CT)—(Centiloid_PET/MRI)	ρ = − 0.39 (*p* < 0.001)*	ρ = − 0.19 (*p* = 0.09)	ρ = 0.13 (*p* = 0.26)	*p* = 0.95

Spearman’s correlation was used to compare (SUVr_PET/CT)/(SUVr_PET/MRI) ratio and Centiloid scale, scan interval, or age. The Mann–Whitney U test was used to compare (SUVr_PET/CT)/(SUVr_PET/MRI) ratio between male and female participants

(Centiloid_PET/CT)—(Centiloid_PET/MRI) was similarly analyzed, showing a significant negative correlation for Centiloid scale (ρ = − 0.39, *p* < 0.001) and a tendency toward correlation for scan interval (ρ = − 0.19, *p* = 0.09) (Table 6).

### Multiple regression analysis of (SUVr_PET/CT)/(SUVr_PET/MRI) ratio and (Centiloid_PET/CT)—(Centiloid_PET/MRI)

The final models identified Centiloid scale and age as significant predictors of (SUVr_PET/CT)/(SUVr_PET/MRI) ratio when using reference regions of the pons, WC, and WCB. Centiloid scale showed strong negative associations (*p* < 0.001 for all), indicating that a higher amyloid burden was associated with a lower (SUVr_PET/CT)/(SUVr_PET/MRI) ratio. Age was a significant positive predictor (*p* = 0.03, 0.02, and 0.01, respectively), suggesting an age-related increase in (SUVr_PET/CT)/(SUVr_PET/MRI) ratio. Scan interval was excluded because it did not provide additional explanatory power in the stepwise multiple linear regression analysis.

In contrast, when using CGM as the reference region, Centiloid scale, age, and scan interval were retained as significant predictors of (SUVr_PET/CT)/(SUVr_PET/MRI) ratio. Centiloid scale again showed a highly significant negative association (*p* < 0.001), scan interval showed a negative association (*p* = 0.03), and age remained a significant positive predictor (*p* = 0.01).

Similarly, both Centiloid scale and age were significant predictors of (Centiloid_PET/CT)—(Centiloid_PET/MRI). Centiloid scale exhibited a highly significant negative association (*p* < 0.001), suggesting that a higher amyloid burden was associated with a smaller Centiloid difference. Age was also a significant positive predictor (*p* = 0.03), indicating an age-related increase in Centiloid discrepancy between PET/CT and PET/MRI.

### Amyloid dynamic PET/MRI

**Fig. 5:**
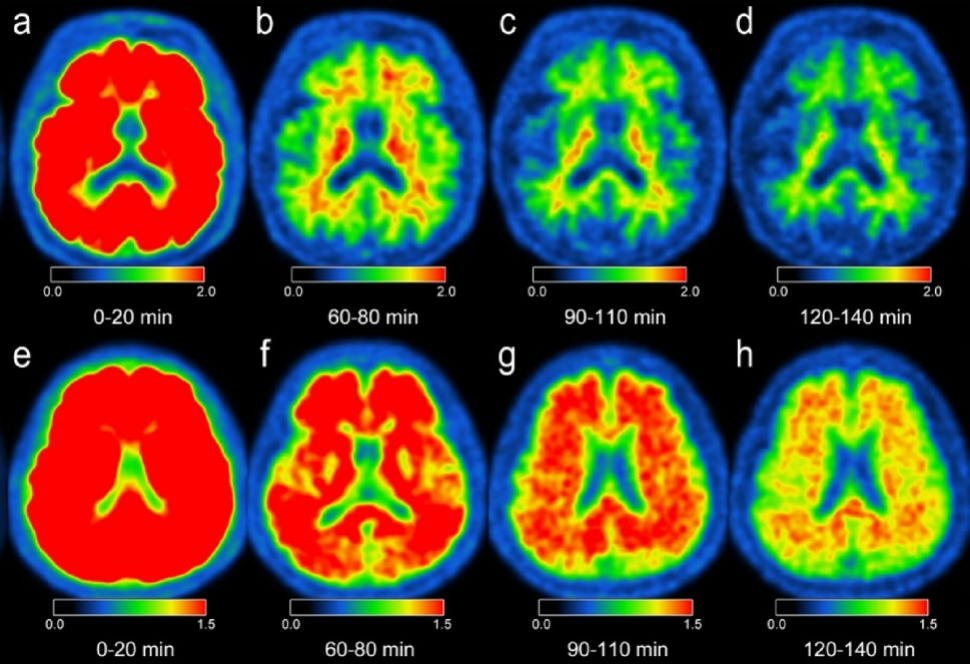
Dynamic amyloid PET/MRI. Dynamic amyloid PET/MRI was performed to minimize the impact of differences between imaging modalities. Representative cases of amyloid-negative (an 85-year-old man; **a**–**d**) and amyloid-positive (a 69-year-old woman; **e**–**h**) subjects are shown. Images were acquired at 0 min (**a**, **e**), 60 min (**b**, **f**), 90 min (**c**, **g**), and 120 min (**d**, **h**) post-injection. Tracer accumulation persists longer in the amyloid-positive case (**h**) than in the amyloid-negative case (**d**)

**Fig. 6:**
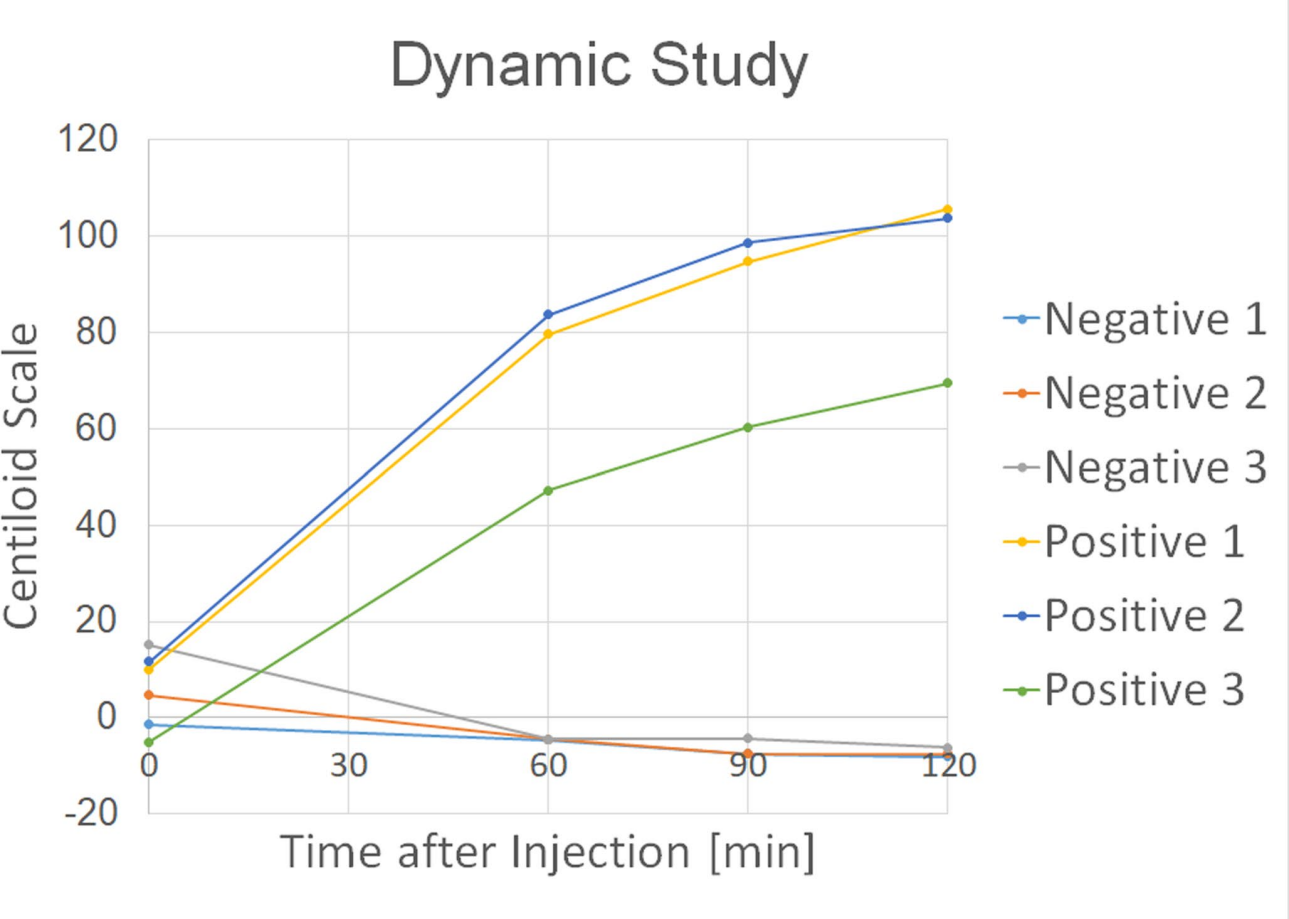
Temporal changes in Centiloid scale values from the additional dynamic PET/MRI study. Amyloid-positive individuals (n = 3) show increasing Centiloid scale values over time, whereas values in amyloid-negative individuals (n = 3) remain stable

Centiloid scale values for each participant are presented in Supplementary Table 2. Representative cases of amyloid-negative and -positive participants are shown in Fig. 5. Chronological changes are illustrated in Fig. 6. Notably, Centiloid scale values among amyloid-positive participants increased over time at 60, 90, and 120 min post-injection, whereas those of amyloid-negative participants remained stable.

## Discussion

Our study compared amyloid PET/CT scans obtained 90 min after administration of ^18^F-flutemetamol with subsequent PET/MRI scans obtained approximately 120 min post-injection. Despite differences in scan timing and imaging modality, high concordance was observed in visual interpretations. Quantitative measurements such as SUVr and Centiloid scale also exhibited high concordance between the two modalities. Visual interpretation and CSF analysis demonstrated high concordance in the diagnosis of amyloid deposition. Despite high concordance of SUVr and Centiloid scale, we observed that Centiloid scale and age were significantly associated with (SUVr_PET/CT)/(SUVr_PET/MRI) ratio and (Centiloid_PET/CT)—(Centiloid_PET/MRI). Regarding SUVr (CGM), scan interval also showed a significant association with (SUVr_PET/CT)/(SUVr_PET/MRI) ratio in addition to Centiloid scale and age.

Visual interpretations indicated minimal variation for each examination conducted 90 and 120 min post-injection in this study. Previous research has demonstrated that images obtained shortly after ^18^F-flutemetamol administration reflect cerebral perfusion because of the high lipophilicity of the tracers [34]. In contrast, images stabilize after 80 min, thereby reducing the influence of imaging start time on visual interpretation [18]. The present findings are consistent with these observations. In the literature, few studies have compared visual interpretations of ^18^F-flutemetamol PET obtained 90 and 120 min post-injection. In contrast, Food and Drug Administration label for ^18^F-florbetaben specifies the scan acquired anytime during an acquisition window ranging from 45 to 130 min post-injection for visual interpretation. However, Centiloid scale increased over time and was most pronounced in those participants with the highest amyloid burden [35]. In a dynamic study of ^18^F-florbetaben, a dual-window (0–30/120–140 min) acquisition with an overall scan time of 50 min provided a good compromise between patient comfort and quantification accuracy, compared with a 140-min acquisition [36].

A significant difference in SUVr or Centiloid scale was evident between PET/CT and sequentially obtained PET/MRI, as shown in Table 3, but the trend differed between SUVr (CGM) and the other metrics. In the low Centiloid scale group, the trend was similar in all participants, but SUVr (CGM) showed no significant difference. In the high Centiloid scale group, SUVr (Pons) was significantly higher on PET/CT, while SUVr (CGM) remained significantly lower on PET/CT, suggesting tracer retention in the cerebral cortices compared with the less affected CGM; this is partly supported by our additional results for amyloid dynamic PET/MRI. However, good concordance in SUVr and Centiloid scale was seen between PET/CT and PET/MRI, probably because normalization to reference regions mitigated these differences effectively. This is consistent with a previous study, which demonstrated that SUVr remained stable from 80 to 170 min after tracer administration [18]. Given that SUVr and Centiloid scale can serve as supportive quantitative metrics, the good concordance despite differences in tracer administration protocols and imaging modalities may provide valuable evidence of their robustness.

Centiloid scale and age are significantly associated with (SUVr_PET/CT)/(SUVr_PET/MRI) ratio and (Centiloid_PET/CT)—(Centiloid_PET/MRI). Our additional amyloid dynamic PET/MRI clearly showed that Centiloid scale increased according to the post-injection delay in the amyloid-positive group, which also supports “retention” of uptake in the cortices of amyloid-positive participants (Figs. 5 and 6). Similar observations were reported in a previous phase 1 study involving ^18^F-flutemetamol [18]. In a recent study using ^18^F-florbetaben PET, “head-to-head” comparison (n = 6) showed a similar trend to our ^18^F-flutemetamol PET protocol: a sequential scan performed at 70–90 min post-injection using PET/CT and at 90–110 min using PET/MRI demonstrated higher SUVR values on PET/MRI [26]. Time-dependent accumulation may reflect both physiological differences in Aβ clearance and the high binding affinity of the tracer in amyloid-positive brains. Meanwhile, the reduction in SUVr and Centiloid scale remained in amyloid-negative participants, which may suggest that retention of amyloid PET tracer will not be lasting.

Beyond the selection of reference regions and scan timing, age was identified as an independent factor influencing the quantitative differences between PET/CT and PET/MRI because of showing a positive correlation with the (SUVr_PET/CT)/(SUVr_PET/MR) ratio and with (Centiloid_PET/CT)—(Centiloid_PET/MRI). Clinically, it is essential to acknowledge these minor yet consistent variations when interpreting scans in borderline cases and when monitoring quantitative values over time across different modalities or protocols. In research contexts, failing to account for age effects can lead to increased variance and weaken the observed treatment or group effects.

This study identified a 98% concordance rate (51 of 52 cases) between results of CSF analysis and visual interpretation using amyloid PET. The best CSF measures showed similar diagnostic accuracies (area under the curve (AUC) 0.93–0.94) to the best PET measures (AUC 0.92–0.93) among both healthy elderly individuals and 34 patients with MCI who developed AD dementia within 3 years (MCI-AD) [37]. Overall percentage agreements between CSF biomarker ratios and ^18^F-florbetaben PET in an MCI population were 87% for Aβ_1–42_/Aβ_1–40_ (*n* = 155) and 88% for pTau181/Aβ_1–42_ (*n* = 94) in a multi-center study [38]. CSF Aβ_1–42_/Aβ_1-40_ correlates more closely with amyloid-PET than Aβ_1–42_ [39]. Combined biomarkers in CSF, particularly pTau/Aβ_1–42_ and Aβ_1–42_/Aβ_1-40_, predicted amyloid-PET result better than Aβ_1–42_ [40]. Regional estimates of Aβ deposition measured with PET offer potential advantages over CSF [41] as well as providing non-invasive detection of amyloid.

Although visual interpretation remains consistent between PET/CT (90 min) and PET/MRI (approximately 120 min), it is crucial for quantitative analyses, especially in research and clinical trials, to incorporate age and scan timing along with reference region as covariates or to standardize them by protocol. This approach helps to address subtle yet systematic variations in SUVr/Centiloid, which is particularly important for cases that are close to threshold values.

This study showed several limitations. First, the use of two different PET scanners (PET/CT and PET/MRI) as well as two different post-injection times (90 min for PET/CT and approximately 120 min for PET/MRI) may have influenced the observed sequential changes in SUVr and Centiloid scale. Amyloid dynamic PET/MRI data clearly demonstrated sequential changes according to the post-injection interval, which likely would have mitigated the influence of PET scanner-related differences. Second, the numbers of amyloid-positive and -negative participants were not balanced. Third, CSF analyses were performed in only a limited number of participants. Fourth, this was a single-center study. In multicenter studies, variations in post-injection timing, in addition to differences among PET scanners, may affect the results and thus require careful consideration. Fifth, head-to-head scans using PET/CT and PET/MRI at the same post-injection delay but on different days were not performed in this study. As far as we investigated, we could not find any such true head-to-head amyloid PET comparisons. However, as described in the previous study [26], our results provide useful information for delayed amyloid PET. Sixth, although quantitative values are influenced by amyloid burden, age, and scan timing, the difference observed in this study was limited, supporting a high concordance between PET/CT and PET/MRI in visual diagnosis and quantitative measurements.

## Conclusion

Our study demonstrated that amyloid PET/CT and sequentially obtained PET/MRI using ^18^F-flutemetamol showed high concordance in both visual interpretation and quantitative measurements, including SUVr and Centiloid scale. However, quantitative values, particularly the Centiloid scale, from delayed-phase PET showed a difference between amyloid-positive and -negative cases, possibly due to differences in the clearance of PET tracer from the cerebral cortices. Age also affects these trends, and scan interval exerted an additional impact when CGM was used as the reference region. While quantitative values may vary depending on Centiloid scale, age, and, in some cases, scan interval, however, visual interpretation agreement was less affected.

## Supplementary Information

Below is the link to the electronic supplementary material.

Supplementary file1 (DOCX 770 kb)

## Abbreviations

Aβ: Amyloid-beta
AD: Alzheimer’s disease
CSF: Cerebrospinal fluid
SUVr: Standardized uptake value ratio
ZTE: Zero echo time
PADNI: Parkinson’s and Alzheimer’s disease dimensional neuroimaging initiative
MCI: Mild cognitive impairment
PD: Parkinson’s disease
OSEM: Ordered subsets expectation maximization
FOV: Field of view
VOI: Volume of interests
WC: Whole cerebellum
CGM: Cerebellar gray matter
WCB: Whole cerebellum and brainstem
ICC: Intraclass correlation coefficient
AUC: Area under the curve
CI: Confidence interval
