# Supplementary material for: Sequential scans of 18F-flutemetamol PET using PET/CT and PET/MRI: influence of amyloid burden, scan interval, and age
Source: Jpn J Radiol. 2025 Dec 10;44(4):685–97. doi: 10.1007/s11604-025-01915-1 (PMC13038638; doi:10.1007/s11604-025-01915-1)
Supplement: Supplementary file 1 — Supplementary file1 (DOCX 770 kb) [file 11604_2025_1915_MOESM1_ESM.docx]

**Supplementary Figure 1** Flowchart of participants in this study.





Flowchart of participants in this study.

#
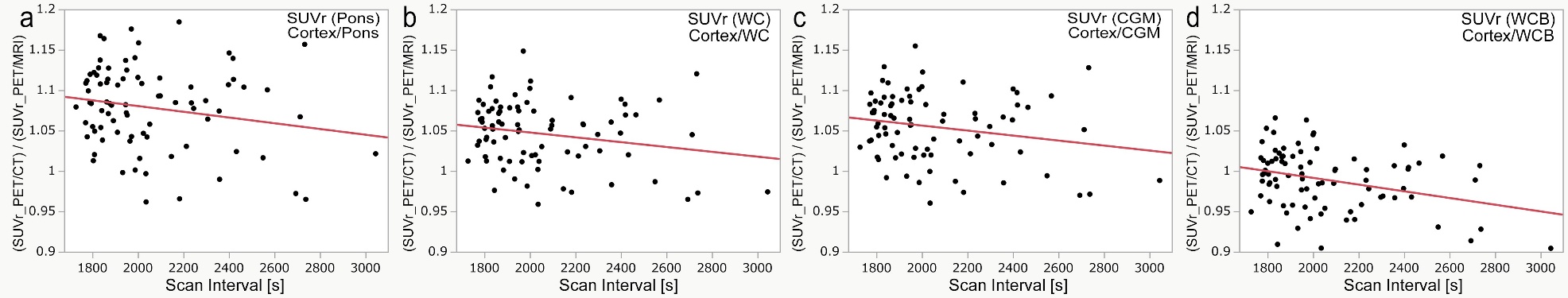
**Supplementary Figure 2** Relationship between ((SUVr_PET/CT)/(SUVr_PET/MRI)) ratio and the scan interval (in seconds) between the two scans.

Relationship between ((SUVr_PET/CT)/(SUVr_PET/MRI)) ratio and the scan interval (in seconds) between the two scans. (a) SUVr using the pons as the reference region (Cortex/Pons), ρ = -0.16, p = 0.16; (b) SUVr using the whole cerebellum (Cortex/WC), ρ = -0.17, p = 0.13; (c) SUVr using cerebellar gray matter (Cortex/CGM), ρ = -0.29, p = 0.01*; and (d) SUVr using the whole cerebellum and brainstem (Cortex/WCB), ρ = -0.17, p = 0.13.

* Statistically significant (p < 0.05).

WC, whole cerebellum; CGM, cerebellar gray matter; WCB, whole cerebellum and brainstem.

# **Supplementary Figure 3** Relationship between ((SUVr_PET/CT)/( SUVr_PET/MRI)) ratios and age (in years).


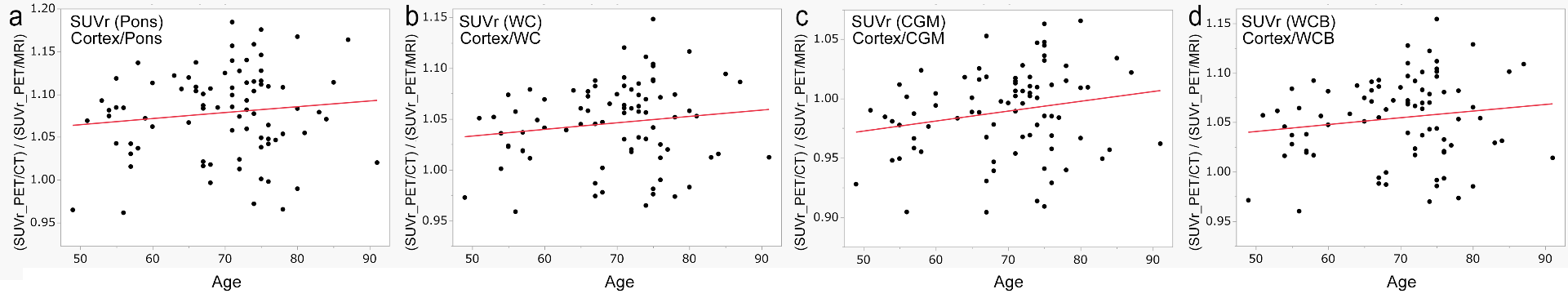


Relationship between ((SUVr_PET/CT)/( SUVr_PET/MRI)) ratios and age (in years). (a) SUVr using the pons as the reference region (Cortex/Pons), ρ = 0.05, p = 0.68; (b) SUVr using the whole cerebellum (Cortex/WC), ρ = 0.10, p = 0.36; (c) SUVr using cerebellar gray matter (Cortex/CGM), ρ = 0.19, p = 0.09; and (d) SUVr using the whole cerebellum and brainstem (Cortex/WCB), ρ = 0.09, p = 0.44.

WC, whole cerebellum; CGM, cerebellar gray matter; WCB, whole cerebellum and brainstem.

# **Supplementary Figure 4** Bland-Altman plot of Centiloid Scale between PET/CT and PET/MRI.


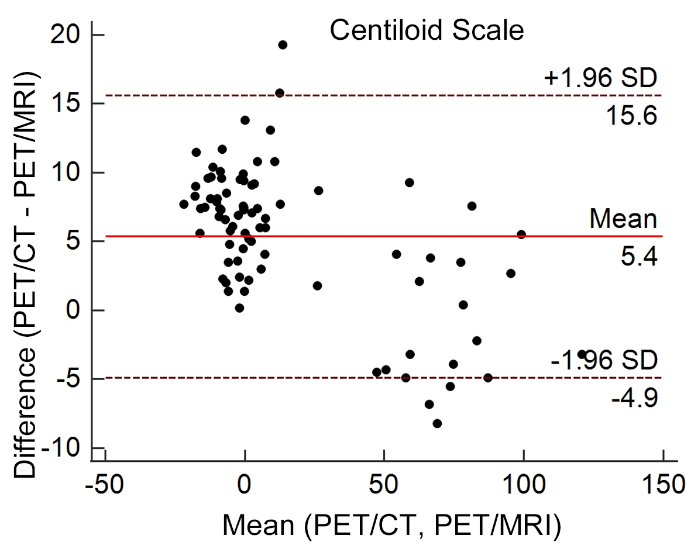


The Bland–Altman plot demonstrated that the Centiloid scale between PET/CT and PET/MRI were mostly within ±1.96 standard deviations.

# **Supplementary Figure 5** Bland-Altman plot of SUVr between PET/CT and PET/MRI.


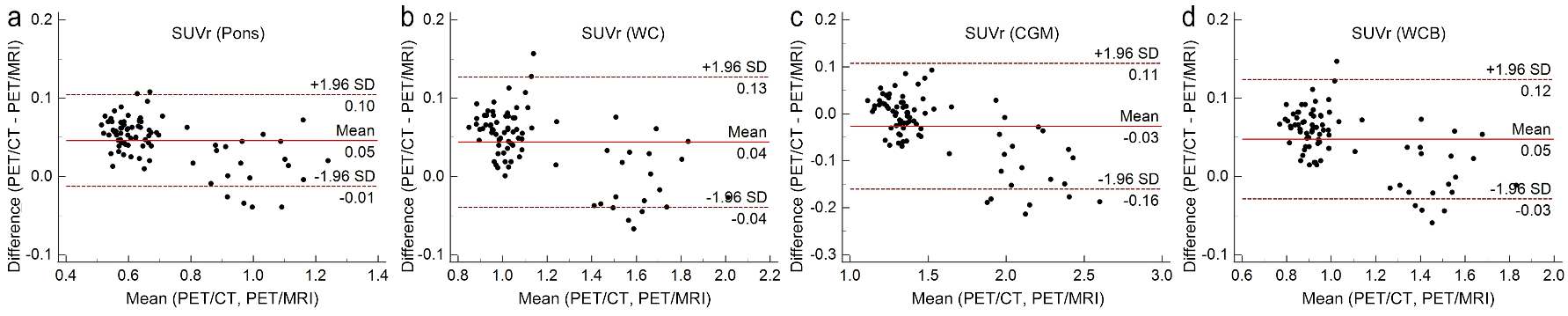


The Bland–Altman plot demonstrated that SUVr values between PET/CT and PET/MRI were mostly within ±1.96 standard deviations. WC, whole cerebellum; CGM, cerebellar gray matter; WCB, whole cerebellum and brainstem.

**Supplementary Table 1. SUV of VOIs**

|  | PET/CT | PET/MRI | *p*-value |
| --- | --- | --- | --- |
| Cortex | 0.86 [0.52, 2.14] | 0.63 [0.38, 1.63] | <0.001* |
| Pons | 1.30 [0.76, 1.99] | 1.00 [0.60, 1.70] | <0.001* |
| WC | 0.78 [0.45, 1.24] | 0.61 [0.37, 0.98] | <0.001* |
| CGM | 0.61 [0.35, 0.98] | 0.45 [0.27, 0.73] | <0.001* |
| WCB | 0.86 [0.50, 1.35] | 0.68 [0.41, 1.07] | <0.001* |

Note that values in square brackets represent the range of each SUV.

* Statistical significance.

WC, whole cerebellum; CGM, cerebellar gray matter; WCB, whole cerebellum and brainstem.

# **Supplementary Table 2**

| Time after injection  [min] | Subject | 1 | 2 | 3 | 4 | 5 | 6 |
| --- | --- | --- | --- | --- | --- | --- | --- |
|  | Age [y],  Sex | 85,  male | 79,  male | 64,  female | 69,  female | 80,  female | 59,  male |
|  | Amyloid | negative | negative | negative | positive | positive | positive |
| 0 | Centiloid scale | -1.5 | 4.7 | 15.2 | 10 | 11.7 | -5 |
| 60 | Centiloid scale | -4.6 | -4.5 | -4.5 | 79.6 | 83.7 | 47.3 |
| 90 | Centiloid scale | -7.6 | -7.6 | -4.3 | 94.8 | 98.7 | 60.3 |
| 120 | Centiloid scale | -8.1 | -7.6 | -6.2 | 105.7 | 103.8 | 69.5 |
